# Nurses' Perceptions of Their Relationships with Informal Carers in Institutional Respite Care for Older People

**DOI:** 10.1155/2013/967084

**Published:** 2013-03-12

**Authors:** Sirpa Salin, Marja Kaunonen, Päivi Åstedt-Kurki

**Affiliations:** ^1^School of Health Sciences, University of Tampere, 33014 Tampere, Finland; ^2^Research Unit, Pirkanmaa Hospital District, 33014 Tampere, Finland

## Abstract

The purpose of this study was to describe nurses' experiences of their collaboration and relationships with family members in institutional respite care for the elderly. The family has a particularly important role in respite care, which is an extension of care provided at home. However no published studies were found on this subject. The data were collected through qualitative interviews (*N* = 22). Content analysis of the nurses' descriptions of their collaboration with family members yielded four main categories as follows: (1) conscious ignoring, (2) attempting to understand the family's situation, (3) hinting at private family matters, and (4) being a friend. The results lend support to earlier findings which emphasize the complexity of relationships between nurses and family carers. A novel finding here is that these relationships may also develop into friendships. Greater emphasis must be placed on primary nursing so that the nurse and informal carer can build up a genuine relationship of trust. If periods of respite care are to help older people and their families to manage independently, it is imperative that nurses have the opportunity to visit their patients at home.

## 1. Introduction

Supporting family carers is a globally recognized objective in elderly care [1]. Nevertheless the development of appropriate services has been a relatively slow process, and the main focus in those services is still on the needs of the care recipient [2].

Finland's population is ageing faster than in any other country in the European Union; latest figures [3] put the number of people aged 65 or over at 941 041, or 17,5 % of the population. The target set in the national old age policy strategy is that 91-92% of the population over 75 should be able to live at home either independently or with the support of social and health care services and/or family members. One of the major elements of the national old age strategy is a support mechanism for informal care, which is governed in Finland by legislation (937/2005). Support is granted mainly on grounds of need for help and on how closely the carer is tied to care provision. Support for informal care includes both a cash benefit (min. 364, max. 729 euros per month) and the right to take three days off each month. The sums earmarked for the benefit vary from one municipality to the next depending on their financial situation, which means that informal carers in different parts of the country are not in an equal position. Informal care is of great importance to the national economy. Estimates for 2006 suggest that without the contribution of carers, an extra 11,600 persons would have needed permanent institutional care. Nevertheless the number of carers receiving government support remains much smaller than the number of those who receive no support at all [4]. The costs of cutting public services for older people have often been borne by informal caregivers [5]. 

Institutional respite care services are provided via both the public and private sector. The cost of service use to the client is the same in either case, and most of the cost is borne by the state. In both cases services are governed by provincial authorities. Most smaller municipalities in the country have chosen to organize respite care as part of their regular long-term health care services, but many larger towns often have dedicated wards in connection with nursing homes.

Carers argue that their frail family members need more services, more support and more guidance, rehabilitation, and training [6]. One of the most important forms of support available to caregivers is respite care. If home care is to succeed and indeed if the carer is to cope with all the demands of care, it is crucial that work is stepped up to develop the range of supportive services available [5]. According to Nies [7], services should all operate in a more “joined-up manner.” This will necessitate new, innovative ways of delivering services to older people. However, no reliable evidence was found that respite care delays entry to residential care or adversely affects frail older people [8].

Collaboration between nursing staff and family members in the home care of older people has recently received increasing research attention, but even so this remains a comparatively under-researched area [9]. This is true particularly in the case of short-term institutional care [10]. Furthermore, most of the work in this field has been done from the carer's point of view.

Earlier studies have shown that caregivers greatly value the opportunity to use respite care services [10–12]. They give them the opportunity for freedom and a normal life for a change and on the other hand release them from concerns about the quality of care. According to Gilmour [10], nurses played a key role in carers' decision on service use. When they had good cooperation with the nurse, carers could trust that their family member would receive proper care; this allowed them to take a break from their care relationship for the duration of respite care. Nurses, for their part, respected these carers' expertise. Caregivers wanted to have a close and personal relationship with nurses [10, 13]. Carers have been found to benefit not only from the break in care provision afforded by respite care, but the support they receive from professionals in terms of information and skills is also important to the continuity of home care [11, 12]. 

Revising family caregiving through an empowerment framework has been shown to guide health professionals in promoting caregiver well-being [14].

Ward-Griffin and McKeever [9] and Jeon [15] have studied the relationship between nurses working in community care and caregivers. Both found that this relationship is a learning process in which both parties learn to work together, albeit at different levels and through different stages. 

Earlier research in acute hospital settings [16, 17] and nursing homes [18, 19] has shown that genuine family involvement in the nursing team is far from a matter of course. The relationship between nurse and family often remains superficial, formal and rather forced [18, 19]. One of the reasons lies in uncertainties about the allocation of powers and responsibilities [20]. The findings are consistent that nurses attach great importance to their collaboration with the patient's or client's family [21–24]. Nurses also seem to agree that family members are the primary sources of information on the patient's as well as the family's situation. The family member's role as a source of emotional and psychosocial support to the patient is also well recognized [21, 24]. On the other hand nurses seem to be less interested in the carer's own situation or in how they are coping [13, 16, 23]. 

A major focus for many earlier studies has been on factors that promote and hamper collaboration between the nurse and carer. One of the key requirements for close collaboration is that family members themselves take the initiative and show an interest in working closely with nurses [10, 19]. Earlier studies have shown that the personal characteristics of both nurses and family members can both promote and hamper collaboration [25]. Hertzberg and colleagues [21] found that nurses actually try to avoid relatives who they find difficult. According to Gilmour [10] it is particularly important for nurses in short-term care to locate themselves in a secondary and supporting care giving role. 

## 2. Materials and Methods

### 2.1. Aim

The study reported here is part of a research project on family nursing underway at the School of Health Sciences, University of Tampere. The aim of this study was to answer the following question: how do nurses perceive their relationships with informal carers of older people in institutional respite care?

### 2.2. Sample

The sample consists of 22 informants, registered nurses (*n* = 3) and practical nurses (*n* = 19), four of whom were men. The informants were employed in two nursing home units specializing in institutional respite care. Furthermore, the interviewees had completed the formal qualifications of primary nurse. After graduation the nurses had worked primarily with older patients on average for 11.5 years (range 1–30 years). Practical nurses in Finland have developed from mere auxiliary workers into health care professionals who work closely with other professionals. They hold a key position in the client-oriented care of older clients [26]. 

### 2.3. Data Collection

The research protocol for the project was submitted for approval to the National Advisory Board on Health Care Ethics in 2002. In this study the data were collected in spring 2005 in a nursing home in one of Finland's largest cities (pop. over 200,000). The nurses were informed about the ongoing research project both orally and in writing. Appointments were made with each of the 22 ( = *n*) nurses and the interviews were conducted during working hours in a conference room outside the ward. The topics of the interview were formulated on the basis of earlier studies [9, 27] (Table 1). The interviews lasted from 55 to 90 minutes (mean 75 min) and they were all conducted by the same researcher. All the interviews were tape recorded and transcribed verbatim. The total research material ran up to 312 pages at 1.5 spacing. 

 Data collection was designed with a view to maximizing information yield, and saturation was reached after interviews with 19 nurses. However, since the final number of participants was not specified in advance [28] and all the nurses wanted to participate in the research, the interviews were continued until all of them had been interviewed.

### 2.4. Data Analysis

**Figure 1:**
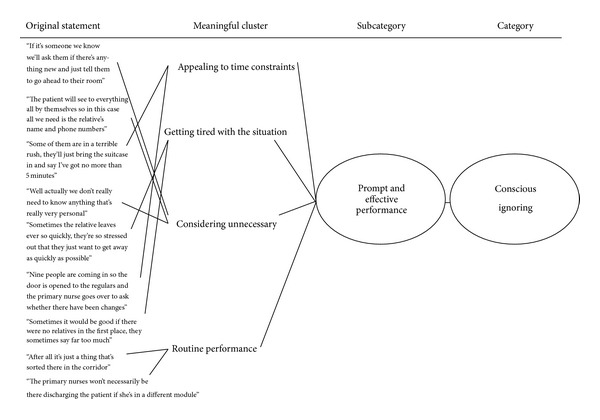
Example of category development in content analysis.

Data analysis consisted of conventional content analysis [29] (Figure 1). First, the research material was read through by one of the researchers several times in order to gain a sense of the whole. The data were then coded verbatim in order to extract key notions. In the process of analysis the researcher made notes of her impressions, thoughts, and preliminary interpretations. A tentative coding system was created by using labels extracted directly from the text. These codes were then grouped into meaningful clusters. At this stage there were still a large number of clusters to facilitate classification. The analysis was continued by combining clusters with similar contents into subcategories, which were given appropriate labels to describe their contents. Subcategories comprising similar contents were finally combined into categories and labelled according to their contents. 

### 2.5. Ethical Issues

The ethical principles of qualitative research that warranted attention in this study were the autonomy and beneficence of nurses. Only very few of the nurses in the sample had previous experience of research interviews and therefore it was considered important to stress to them that participation was voluntary and that all the data collected would be handled in confidence and anonymously. All interviewees were in the employ of the same organization and they also knew one another well. To minimize any risk that the interviewees might be identified, they were interviewed alternately from both wards. It is for this same reason of anonymity that the original interview excerpts quoted in this article make no reference to the respondent's gender or job position [30]. 

## 3. Results

**Figure 2:**
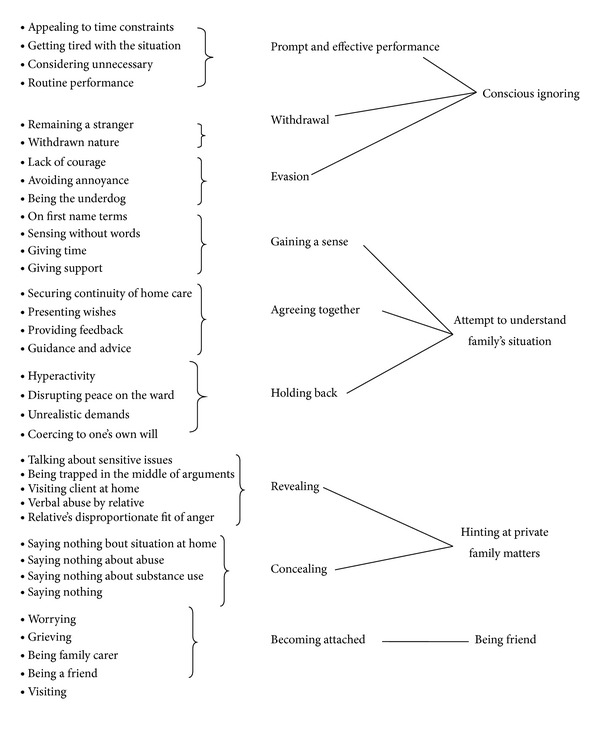
Collaborative relationship between nurse and family carer in respite care for elderly patients.

The interviewees described their relationship with carers via four categories (Figure 2).

### 3.1. Conscious Ignoring

Conscious ignoring (Figure 1) in the nurse-caregiver relationship was reflected in the mutual sense that there was not enough time for interaction. Family carers did not seem to have the time to get genuinely involved in matters concerning their relative. Nurses presumed that carers were simply so exhausted that the only way they could try to cope was to skate over problems as quickly as possible. Nurses, for their part, were also extremely pressed for time in their job. Sometimes up to nine new patients were being admitted at the same time, and on top of that the previous group of patients was being discharged. Especially in the case of regular clients it was felt that there was no point wasting precious time talking to family members. The relationship was described as a matter of routine, with communication effectively confined to asking whether there was anything new or special that the nurses needed to know (Figure 1).

*“…I mean obviously the first time round it's important to explain how these things work, but when they've been here a few times things go more quickly … all you have to do is wish them a good break.” *

Conscious ignoring was also reflected in a mutual tendency of withdrawal as well as in the nurse and carer remaining strangers to each other. Nurses seemed to think that family members were withdrawn and reluctant to have any closer contact with the nurses. Some nurses said they positively loathed the idea of asking carers how things were at home; this was tantamount to prying into private matters. The nurses' tendency of withdrawal was manifested in their lack of courage to go up to the carers and talk to them. Some were reluctant in their capacity as primary nurses to assume responsibility for their patient's overall care. As a result they also developed no caring relationship with the family.

*“…We also get these fast-track types in, quite clearly. The client is admitted, they leave in a fortnight's time and absolutely nothing at all happens during this period… [22]” *

One of the strategies of consciously ignoring others was that of evasion. Nurses took the view that family members lacked the courage to approach them and speak to them. Sometimes they would slip into the patient's room without anyone even noticing. Some of the nurses admitted to their own reluctance to approach family members. Avoidance was a conscious strategy most particularly vis-à-vis demanding daughters or wives. Nurses were apprehensive when they knew a difficult family member would be visiting the ward and preferred to keep away when they did.

*“…I mean we just can't do all of it, can we. I mean this wife she spent hours on end here every day and every day there was something she … there was always something that was wrong. That's not at all amusing. Really you don't want to go, you don't want to be there when they come, if I'm completely honest to you… [13]”*

### 3.2. Attempting to Understand the Family's Situation

Nurses also wanted to foster a collaborative relationship with carers by trying to understand the family's situation. This required an active interest on the part of nursing staff as well as a keen grasp of situations because carers rarely took the initiative to approach nurses. The carer did not necessarily have to say anything about their situation at home, but the nurse would simply observe their facial expressions, gestures, and the whole way they carried themselves. Nurses said they could infer much more about the family's situation from the patient's behaviour and their extraordinary caring demands than they could from any verbal accounts. Many carers were also said to have a deeply felt need to give vent to all the pent-up emotions caused by their current situation. That, however, required a close relationship of trust and confidence, which the nurses described as being on first-name terms. In this situation nurses felt they were capable of offering support to carers.

*“…I'm sure they never say it straight out that they're exhausted,…often there's this absolutely unnecessary fuss, somehow you don't necessarily hear them say it, it's more a matter of observing their behaviour and the rest of it, seeing their fatigue and exhaustion...” *

The attempt by nurses to understand the family's situation was also reflected in their seeking to come to agreement with carers on various day-to-day decisions. Both were keen to ensure the continuity of home care as well as institutional respite care. Many carers had written instructions for nurses, who had no objection to this. Nurses recognized that the carers were competent care providers. Nurses rarely gave any advice to carers, or at least they did not feel they were doing so.

*“…the thing is they really have a way of organizing these things. Sometimes it's quite unbelievable, I mean without any  ... well I suppose you do become a nurse of sorts if you look after your spouse at home…”*

For the most part the behaviour of carers during their visits on the ward was regarded as appropriate, which helped to foster better collaboration. They helped their relatives with routine everyday activities, which made the nurses' jobs easier and was therefore gratefully accepted. Sometimes it was necessary to hold back the carer who could not see the difference between the ward and their own home. Overactive carers might delve into such jobs as reorganizing the ward's refrigerator and disturbing other patients' meals simply by making a loud presence of themselves.

*“…I mean honestly this relative was absolutely impossible. She got involved in everything…every afternoon the wife appeared and began fussing about and complaining and she must have had a very hard time of it herself...” *

### 3.3. Hinting at Private Family Matters

Hinting at private family matters was reflected in both disclosing and concealing information about family life. Sometimes family members would readily disclose even very sensitive and private information, without any sense of shame. Excessive drinking and the abuse of medication were major causes of concern for carers. Periodic admissions for respite care were often like a short detox programme, and all the carer wanted was to see the patient's human dignity restored. Both upon admission and at discharge carers were particularly concerned about how the patient would cope at home and how they themselves would manage. According to the interviewees carers did not expect their problems to be resolved; all they wanted was someone they could trust and who would listen to them and encourage them. Hinting at private family matters required a collaborative relationship based on mutual trust, the foundations for which were created upon admission to respite care.

* “…actually it's pretty important the admission situation… if it's all a bit unimpressive and you just try to get through it all quickly, then obviously the impression won't be very good...” *

Sometimes nurses found themselves caught up in the middle of arguments between family members. Tact was required especially in situations where family members were critical of each other behind each other's backs. Nurses were particularly unhappy to find themselves in situations where they were being persuaded to side with one or the other party. Many of these arguments had to do with decisions of permanent institutionalization against the patient's will. Family members would try to persuade nursing staff to support their view by constantly calling and visiting them to make their arguments. They refused to cooperate and eventually wore down all the parties.

*“…it's really difficult and really annoying and really gets on your nerve and you feel you have this band around your head all the time, clamping you from first thing in the morning, oh no here we go again with this same old... [21]”*

Given the behaviour of family members on the ward, nurses said they sometimes found themselves wondering whether the patients were safe at home. Sometimes a family member would become verbally abusive against the nurse for the slightest of reasons, clearly disclosing an easily agitated temperament. Bruises and a tendency for older patients to become emotional rather too easily turned the nurses' thoughts to the possibility of abuse at home: this might include financial exploitation or general maltreatment. The nurses notified the social worker of any obvious instances of abuse. The matter was never raised with the family members or the patients themselves; this remains a firm taboo. 

Many of the interviewees had visited their patient's home, either to return something that had been forgotten on the ward or to check that conditions at home were such that the patient could indeed cope. The best way to get to know the patient's family, the nurses felt, was to see how they lived at home: seeing their home told them more than any description ever could. Most nurses felt that they did not know enough about the patient's situation at home. Therefore it was impossible for them to have a very clear idea of how respite care fitted into the rest of the family's life.

*“…it gives you a completely different perspective when you've been round to see for yourself… I've seen the physical environment where they live… I mean… it says nothing when you learn that they live in a block of flats. But when you've seen that flat, it says a great deal about that person…” *

According to the nurses' accounts hinting at private family matters was not easy for either party. Family members concealed the real situation at home by saying nothing about their problems, and nurses did not always dare ask unless the initiative was taken by the carer. Very often hidden problems only surfaced at meetings of the multiprofessional health care team. There might have been indications of abuse, but because of the threat of disclosure family members may have decided to take the patient home early and never return. Sometimes carers also opted to say nothing about the patient's abuse of medication and excessive drinking, and these problems would only become apparent with the appearance of withdrawal symptoms, nausea, and irritability.

It was clear from the nurses' accounts that they, too, concealed things that had happened during the patient's stay. They would do so when they knew or suspected that the carer would have objected or been annoyed. Nurses respected the patient's wishes by allowing them to smoke, for example. Referring to their professional competence, nurses had a different assessment of the situation than the carer who imposed restrictions. However the disclosure of whatever family members had forbidden undermined the credibility of nurses as well as the relationship of trust between nurse and carer. Sometimes the clients themselves wanted to conceal what had happened during their stay from their relatives and insisted that the primary nurse say nothing to family members. 

### 3.4. Being a Friend

Nurses and carers sometimes became friends who shared a mutual affection. In long-term relationships the nurse became part of the patient's family. Occasionally nurses became friends with the patient only and worried about how their friend was doing when they were at home. The death of a patient was often a heavy blow even to nurses, and they would grieve together with family members. Friendships sometimes extended beyond the patient's death. During their time off nurses might visit their clients and attend family celebrations. Friendship was also seen in patients or carers maintaining contact with the nurse during periods when they were at home. On the one hand nurses were worried about how their friendship affected their professionalism. At least initially, friendship required working as a primary nurse. Once they had become friends, nurses were reluctant to continue as the patient's primary nurse because they were wary of becoming too closely involved in the patient's and family's private life. Conflicts and disagreements between family members and patients were particularly awkward in situations in which it was thought that the nurse's professionalism may be compromised.

*“…I've been trying to say this to these primary nurses that when they've been with these long-term patients who've been here for a long time … that they remember the limits, that you can't keep it up if you get involved …. You also get these situations where you have to be on the client's side, if you really become a friend with family members, you have to be able to retain your professionalism ….” *

## 4. Discussion

The nurses' descriptions of their cooperation with carers resulted in four main categories: conscious ignoring, attempting to understand the family's situation, hinting at private family matters, and being a friend. The results describe tendencies in the collaborative relationship, rather than distinct strategies adopted by the nurses. It was thus possible for a single nurse to show aspects of several categories in his or her dealings with the carer. 

One important goal of respite care nurses is to support the carers, which requires both parties' willingness to work together [15]. According to the results obtained in this study, nurses sometimes even consciously ignored carers, making collaboration impossible. It was particularly difficult or even impossible for nurses to focus on the carers during the patients' admittance into respite care, as they were busy with the leaving patients. In these situations nurses sometimes chose to handle the interaction as fast as possible, especially if they were already familiar with the patient. While this is understandable, such behavior does not encourage carers to talk to the nurses on a nonsuperficial level. They have no chance to talk about the events of the home care or share their wishes about the contents of the respite period. Because of this, it can be difficult for carers to trust the patient will receive quality care. According to earlier studies [10, 13], carers do wish to have a close and personal relationship with the nurses.

One reason for the failure to form a collaborative relationship may be the idea that the family's personal issues do not concern the respite care staff, even when they are related to the patient. The staff focuses solely on the patient's physical needs, failing to provide holistic care or consider the carer's needs. Respite periods and home periods follow each other as separate phases in the family's life. Nurses who value their profession must seek to understand the reasons behind carers' dissatisfaction rather than avoiding them and situations they find difficult. Carers' despair and disappointment at their perceived lack of fortitude can be a reason for their dissatisfaction, which they sometimes vent at nurses. Disagreement between nurses and carers is not uncommon [20, 21]. The nurses interviewed in this study felt themselves helpless before demanding carers [21] and had no other way of handling the situation than avoiding them. Support from colleagues and facing difficult situations together is the professional way of dealing with such problems, which benefits carers as well.

However, this study also found that nurses would consciously build their collaborative relationship with carers by spending time with them alone [15, 25]. Nurses were interested in the carers' coping and tried to support them in various ways. Nurses need to be able to approach carers who do not discuss their thoughts on their own initiative. This enables them to share personal and difficult matters with the nurses. Building trust and a collaborative relationship takes time, which is why the primary nurse should not be changed. This study also found that setting and clarifying boundaries during the respite period was also a form of supporting carers, though nurses found it difficult and time-consuming. Contrary to the findings of earlier studies, nurses welcomed carers' participation in treating their patient during respite periods [19, 20].

Another form of collaboration between nurses and carers identified in this study is hinting at private family matters, which included both concealing and revealing difficult issues in the family's life. The results can be used to consider the reasons that allow or prevent carers from speaking openly [15]. According to Ashworth and Baker (2000) [11], both carers and patients require guidance, education, and advice on the use of respite care services to enable them to fully benefit from them. One reason for the concealing discussed by the nurses can simply be the carers' inability to receive proper help in dealing with the underlying causes of their problems during respite periods.

The nurses in this study found situations involving family tensions difficult, which is consistent with earlier studies [9, 10, 15, 21]. One such situation the nurses described was the patient instructing how much the nurses were allowed to tell about the patient's issues to their family. In other cases family members would try to make the nurses choose sides in family arguments. The patient's inability to cope at home and potential institutionalization, even against the patient's will, were difficult and sometimes exhausting topics to the nurses. In situations such as these, neither the nurses nor the carers should be left alone. A multiprofessional team is required to handle the matter. On the other hand, if nurses notice the patient is being mistreated, they should not leave the issue only to other professionals such as social workers, as this leaves the family without nursing support (cf. [21]).

In cases where nurses stopped acting as the family's primary nurse after befriending them, the patient and carer often found it difficult to understand the reason for their decision. It is paradoxical that the nurse who best knows the family's situation and has won their trust should withdraw out of fear of not being able to properly perform their duties. Nurses in such situations should carefully consider if they are truly helping the family by stepping aside. It is not surprising that nurses become friends with the families they work with, as the relationship often lasts for years.

### 4.1. Trustworthiness

The main difficulty with the method of content analysis lies in how well the researcher succeeds in extracting meaningful categories from the data and in demonstrating how they tie in with that data [29]. The analysis is described in this article in such a way that the reader can trace its various stages. 

According to Lincoln and Guba (1985) [28] credibility refers to the truthfulness of the results and depends among other things on the commitment of the researchers to the study. One of the researchers in this study has extensive experience of working with older patients as well as of the interview method used, while another has a strong background in theory building in the field of family nursing. The clearest indication of our success in achieving the trust of the informants was that they all wanted to participate in the research. Transferability refers to the applicability of the results in other, similar contexts. The informants only represented the staff of two wards specializing in respite care from Southern Finland. However, similar care is also provided in home settings as well as on ordinary nursing home wards especially in rural areas.

Dependability refers to the presence of human variation. The interview situations here were made as similar to each other as possible, since the nurses were interviewed during working hours in an environment that was familiar to them. It is possible that the time pressures on the ward adversely affected the nurses' ability to concentrate on the interview. Confirmability, as an element of reliability, means that the results are drawn from the material rather than based on the researchers' personal views. 

## 5. Conclusions

According to our results, the following conditions are necessary for the nurse and carer to reach a collaborative relationship.

- Forming a collaborative relationship requires time, which means the unit should not admit and discharge respite care patients at the same time. The focus must be on the family, rather than the organization. This allows for continuity between the care given at home and the care at the unit. Another important form of creating a collaborative relationship and understanding of the family's situation is the nurse visiting the family at home, particularly at the beginning of the care relationship.
- Both parties require education. Information aimed at carers must include matters related to the contents of the care and their own role in influencing its quality. Nurses need to be trained in recognizing their own starting point in difficult family matters and the nurse's role in holistic professional caregiving. Group-based job supervision and support from colleagues are important methods of ensuring the nurses are able to cope at their jobs.

## Figures and Tables

**Table 1:** Topics of interviews with nurses.

Interview themes	
(i) Your education and experience in the nursing care of the elderly	
(ii) Describe your interaction with informal carers	
(a) upon admission to respite care	
(b) during respite care	
(c) upon discharge from respite care	
(d) during home care	
(iii) Describe your own thinking about working with the family of elderly patients	
